# Origin and spatiotemporal dynamics of the peroxisomal endomembrane system

**DOI:** 10.3389/fphys.2014.00493

**Published:** 2014-12-16

**Authors:** Vladimir I. Titorenko, Richard A. Rachubinski

**Affiliations:** ^1^Department of Biology, Concordia UniversityMontreal, QC, Canada; ^2^Department of Cell Biology, University of AlbertaEdmonton, AB, Canada

**Keywords:** peroxisome, lipid metabolism, reactive oxygen species, peroxisome biogenesis, endoplasmic reticulum, peroxisomal endomembrane system, development, aging

The peroxisome is an organelle with essential roles in lipid metabolism, maintenance of reactive oxygen species homeostasis, and anaplerotic replenishment of tricarboxylic acid cycle intermediates destined for mitochondria (Islinger et al., 2012; Beach and Titorenko, 2013; Wanders, 2014). Peroxisomes constitute a dynamic endomembrane system. The homeostatic state of this system is upheld via two pathways for assembling and maintaining the diverse peroxisomal compartments constituting it; the relative contribution of each pathway to preserving such system may vary in different organisms and under various physiological conditions. One pathway begins with the targeting of certain peroxisomal membrane proteins to an endoplasmic reticulum (ER) template and their exit from the template via pre-peroxisomal carriers; these carriers mature into metabolically active peroxisomes containing the entire complement of membrane and matrix proteins (Titorenko and Rachubinski, 2009; Hu et al., 2012; Tabak et al., 2013). Another pathway operates via growth and maturation of pre-existing peroxisomal precursors that do not originate from the ER; mature peroxisomes proliferate by undergoing fission (Nuttall et al., 2011; Hettema et al., 2014; Knoops et al., 2014). Recent studies have uncovered new roles for the peroxisomal endomembrane system in orchestrating important developmental decisions and defining organismal longevity (Titorenko and Rachubinski, 2004; Dixit et al., 2010; Beach et al., 2012). This Frontiers Research Topic is focused on the advances in our understanding of how evolutionarily distant organisms coordinate the formation, maturation, proliferation, maintenance, inheritance, and quality control of the peroxisomal endomembrane system and how peroxisomal endomembranes communicate with other cellular compartments to orchestrate complex biological processes and various developmental programs from inside the cell. Veenhuis and van der Klei (2014) provide insights into the mechanisms underlying the biogenesis of early peroxisomal precursors that do not arise from the ER. In the yeast *Hansenula polymorpha*, these vesicular precursors undergo multistep maturation into metabolically active peroxisomes only after acquiring the peroxin Pex3; the subsequent import of membrane and matrix proteins into such Pex3-containing peroxisomal precursors drives their multistep conversion into mature peroxisomes. Agrawal and Subramani (2013) discuss the relationship between two alternative routes of peroxisome biogenesis. One route involves the *de novo* formation of peroxisomes from an ER template, while the second involves the growth and division of pre-existing peroxisomes. The authors suggest that both routes operate simultaneously in organisms across phyla and propose a model that integrates the two routes into a single pathway for peroxisome assembly and maintenance. The model posits that a balance between progression rates of the two routes is modulated by various intracellular and extracellular signals; such modulation enables to preserve the peroxisomal endomembrane system under various physiological conditions. Kim and Mullen (2013) review the diverse ways through which the peroxin Pex16 can function to preserve the peroxisomal endomembrane system in such evolutionarily distant organisms as the yeast *Yarrowia lipolytica*, the plant *Arabidopsis thaliana*, and mammals. They dissect the mechanisms by which this peroxin orchestrates both routes of peroxisome biogenesis, i.e., the route of *de novo* formation of peroxisomes from an ER template and the route of growth and division of pre-existing peroxisomes, in these organisms. To explore the relationship between peroxisomes and the ER, Barton et al. (2013) concurrently visualized both organelles in living *A. thaliana* plants expressing differently colored peroxisome- and ER-targeted fluorescent proteins. The authors provide evidence that, although peroxisomes can be found in close contact with the ER, no luminal continuity exists between the two organelles. Mohanty and McBride (2013) evaluate evidence that the recently discovered vesicular flow from mitochondria to peroxisomes plays important roles in the assembly, maintenance, metabolic functions, and signaling activities of the peroxisomal endomembrane system in mammalian cells. They outline the molecular mechanisms underlying such a vesicular coupling of mitochondria to different compartments of the peroxisomal endomembrane system. Hasan et al. (2013) explore the molecular dynamics of a multistep process for protein import into the matrix of peroxisomes. They discuss the recent advances in our understanding of the mechanisms underlying the formation of a receptor/cargo-complex in the cytosol and its subsequent docking at the peroxisomal membrane, the translocation of the cargo protein across the membrane and its release into the peroxisomal matrix, and the recycling of receptor molecules. Ast et al. (2013) discuss the molecular mechanisms by which isoforms of proteins known to be peroxisomal can also be actively sorted to the cytosol, mitochondrion, nucleus, or plastid. The authors hypothesize that such dual sorting of peroxisomal proteins within a cell could have an important role in extending the metabolic capacity of peroxisomes in response to specific changes in cell physiology and/or environmental conditions. Kunze and Hartig (2013) explore how different intermediates of the glyoxylate cycle are transported across the peroxisomal membrane and how individual enzymes of this cycle are distributed along both sides of the membrane. They suggest that the efficient and selective transport of glyoxylate and other metabolites across the peroxisomal membrane may be essential for the high functional adaptability of peroxisomes. Fujiki et al. (2014) discuss the multicomponent protein machineries that in mammalian cells orchestrate the assembly of membrane proteins in the peroxisomal membrane and the import of matrix proteins across this membrane. Nordgren et al. (2013) examine the mechanisms by which a selective degradation of dysfunctional or excessive peroxisomes in a mammalian cell enables it to maintain a healthy population of peroxisomes. They discuss the evidence supporting the essential contribution of defects in peroxisome degradation to human disease. Van Veldhoven and Baes (2013) review how mutations impairing peroxisome biogenesis affect organismal size, development, and longevity in various invertebrate and vertebrate models, including nematodes, fruit fly, zebrafish, and mouse. Their analysis implies that a reduced size at birth, delay in development, and shortened lifespan are the most common features of different peroxisome biogenesis deficiencies. Maruyama and Kitamoto (2013), as well as Peraza-Reyes and Berteaux-Lecellier (2013), explore mechanisms underlying the essential roles of peroxisomes in the morphogenetic program initiated by physical damage to hyphae, biosynthesis of biotin, and sexual development in fungi.

## Conflict of interest statement

The authors declare that the research was conducted in the absence of any commercial or financial relationships that could be construed as a potential conflict of interest.
